# Personalizing Treatment in IBD: Hype or Reality in 2020? Can We Predict Response to Anti-TNF?

**DOI:** 10.3389/fmed.2020.00517

**Published:** 2020-09-02

**Authors:** Raja Atreya, Markus F. Neurath, Britta Siegmund

**Affiliations:** ^1^Department of Medicine, Medical Clinic 1, University Hospital Erlangen, University of Erlangen-Nürnberg Erlangen, Erlangen, Germany; ^2^Deutsches Zentrum Immuntherapie (DZI), Erlangen, Germany; ^3^The Transregio 241 IBDome Consortium, Erlangen, Germany; ^4^The Transregio 241 IBDome Consortium, Berlin, Germany; ^5^Medizinische Klinik m. S. Gastroenterologie, Infektiologie und Rheumatologie, Charité - Universitätsmedizin Berlin, Corporate Member of Freie Universität Berlin, Humboldt-Universität zu Berlin, Berlin, Germany; ^6^Berlin Institute of Health, Berlin, Germany

**Keywords:** inflammatory bowel diseases, anti-TNF, response, prediction, personalized medicine

## Abstract

The advent of anti-TNF agents as the first approved targeted therapy in the treatment of inflammatory bowel disease (IBD) patients has made a major impact on our existing therapeutic algorithms. They have not only been approved for induction and maintenance treatment in IBD patients, but have also enabled us to define and achieve novel therapeutic outcomes, such as combination of clinical symptom control and endoscopic remission, as well as mucosal healing. Nevertheless, approximately one third of treated patients do not respond to initiated anti-TNF therapy and these treatments are associated with sometimes severe systemic side-effects. There is therefore the currently unmet clinical need do establish predictive markers of response to identify the subgroup of IBD patients, that have a heightened probability of response. There have so far been approaches from different fields of IBD research, to descry markers that would empower us to apply TNF-inhibitors in a more rational manner. These markers encompass findings from disease-related and clinical factors, pharmacokinetics, biochemical markers, blood and stool derived parameters, pharmacogenomics, microbial species, metabolic compounds, and mucosal factors. Furthermore, changes in the intestinal immune cell composition in response to therapeutic pressure of anti-TNF treatment have recently been implicated in the process of molecular resistance to these drugs. Insights into factors that determine resistance to anti-TNF therapy give reasonable hope, that a more targeted approach can then be utilized in these non-responders. Here, IL-23 could be identified as one of the key factors determining resistance to TNF-inhibitors. Growing insights into the molecular mechanism of action of TNF-inhibitors might also enable us to derive critical molecular markers that not only mediate the clinical effects of anti-TNF therapy, but which level of expression might also correlate with its therapeutic efficacy. In this narrative review, we present an overview of currently identified possible predictive markers for successful anti-TNF therapy and discuss identified molecular pathways that drive resistance to these substances. We will also point out the necessity and difficulty of developing and validating a diagnostic marker concerning clinically relevant outcome parameters, before they can finally enter daily clinical practice and enable a more personalized therapeutic approach.

## Introduction

Inflammatory bowel diseases (IBD) encompasses chronic inflammatory disorders of the gastrointestinal tract whose phenotypic entities mainly comprises Crohn's disease (CD) and ulcerative colitis (UC) (1, 2). These chronic, relapsing, and remitting diseases are characterized by intestinal inflammation and epithelial injury, causing lifelong morbidity (3). Both IBD subtypes are progressive conditions that can lead to bowel damage and disability, having a major impact on an individual's quality of life. Furthermore, ongoing inflammatory activity is causative for occurrence of strictures, fistula, abscesses (1), as well as heightened incidence of colitis-associated neoplasia (4). Optimized anti-inflammatory therapy is therefore essential in the management of IBD patients.

Growing insights into underlying immunopathogenic mechanisms of IBD have led to the advent of targeted therapies, which selectively inhibit crucial mediators of the inflammatory process (5). The first class of biological therapies approved for the treatment of IBD patients were agents inhibiting the pro-inflammatory cytokine tumor necrosis factor (TNF). This substance class encompasses the chimeric monoclonal antibody infliximab, the monoclonal human antibody adalimumab, corresponding infliximab and adalimumab biosimilars, the fully human monoclonal antibody golimumab, and the PEGylated humanized Fab' fragment certolizumab pegol (6). These inhibitors of TNF are applied for induction and maintenance therapy and have made a major impact on our existing therapeutic algorithms. Their advent and the following introduction of targeted therapies (anti-alpha4beta7 integrin inhibitor vedolizumab, anti-IL-12/IL-23p40 antibody ustekinumab and JAK-inhibitor tofacitinib) have helped us to shift current therapeutic strategies toward achievement of deep and prolonged clinical and endoscopic remission, aiming for prevention of complications and halting the progressive course of disease, improving the quality of life of IBD patients (7).

However, depending on the duration of anti-TNF treatment and the outcome parameters chosen, approximately one third of treated patients do not demonstrate response to therapy (primary non-response). Available data indicate that primary non-response should not be assessed prior week 8–12 after initiated therapy (8). Furthermore, 30–50% of initial responders are prone to loose response to therapy in the course of anti-TNF treatment (secondary non-response). A review of studies evaluating loss of efficacy and requirement of infliximab dose intensification, estimated that the annual risk for loss of response to infliximab is ~13% per patient-year of treatment (9).

There is therefore an urgent clinical need to establish predictive markers of response to identify the subgroup of IBD patients, which have a heightened probability of response to anti-TNF therapy. Such an approach would enable us to prevent a delay of initiating an effective treatment, create a substantial benefit for the patients via selection of the most appropriate agent for rapid response to therapy and improved quality of life (10–13). Treatment with a beneficial therapy also reduces the risk of being exposed to potential systemic side effects of an ineffective therapy. Although anti-TNF agents are generally well-tolerated in clinical practice, they have been shown to increase the susceptibility to serious infections (14), possibly melanoma skin cancer (15), and treatment-related complications, such as lupus-like syndromes or allergic reactions.

Recent cost analyses also identified anti-TNF antibodies as the main cost driver in IBD patients, necessitating the need for predicative biomarkers to enable health-economic sound use of these substances (16, 17). Reliable biomarkers predicting likelihood of therapeutic success to subsequent anti-TNF therapy, would allow utilization of a personalized medicine concept with optimized use of this substance class, providing a substantial benefit for the treated IBD patient (13).

In the following, findings from different fields of research to identify predictors to anti-TNF treatment are discussed. Therapeutic drug monitoring studies, which assessed the influence of trough levels and anti-drug antibody formation on therapeutic response were not considered in this review, as we only selected predictive markers which had to be measured before initiation of anti-TNF therapy.

Potential markers were derived from insights into disease-related and clinical factors, blood and fecal markers, molecular tissue expression, immunogenicity, previous therapies, pharmacogenomics, microbial, and metabolite markers, as well as blood and stool derived parameters.

Utilization of theses markers will hopefully lead to a more strategic approach of patient selection before initiating anti-TNF therapy in IBD. Furthermore, mechanisms underlying the failure to respond to anti-TNF therapy are not completely understood. An improved understanding of molecular resistance mechanisms would similarly be essential to optimize personalized medicine approaches in IBD (10).

## Patient and Disease Related Predictors to Anti-TNF Therapy

Several patient and disease related factors have been described to be associated with treatment response to anti-TNF therapies.

### Age, Gender, Weight

On the one hand, younger age at initiation of therapy has been implied to predict better primary response to therapy in CD (18–20) and UC (21), but on the other hand several studies have not been able to demonstrate any relationship between age and therapeutic success (22–27). Similarly, contradicting data have also be described for gender, as single reports indicated better primary response in male CD (28) and female UC patients (25), but the majority of studies did not find any association (19, 22, 26, 27, 29, 30). Inconsistent results have also been obtained for correlation between weight of the anti-TNF treated patient and primary therapeutic response (13). Pooled analysis of individual participant data from clinical trials of infliximab in IBD did not demonstrate that obesity led to worse therapeutic response (31). Altogether, none of the stated patient related factors can be clearly associated with response to anti-TNF therapy.

### Smoking

From all environmental factors that have been described to affect the disease course in IBD patients, smoking has been identified as one of the most influential. Smokers with CD have a more complicated disease course and discontinuation led to better outcomes (32–34). Although, some studies have indicated worse outcomes of anti-TNF treated smoking CD patients in comparison to non-smokers (35, 36), two meta-analyses found no effect of smoking on primary effectiveness of infliximab in CD patients (37, 38). In UC, smokers have reduced colectomy rates, less primary sclerosing cholangitis and less back-wash ileitis than never smokers (39). In UC, few studies do (25) and most studies do not implicate influence of smoking on anti-TNF primary efficacy (21, 29, 30, 40).

### Disease Duration and Location

In patients with CD, shorter disease duration has been repeatedly described to predict higher responsiveness to anti-TNF drugs. In *post hoc* analyses of phase 3 clinical trials, patients with disease duration below 2 years had significantly better primary response rates to adalimumab (41) and certolizumab pegol (42) than those with long-standing disease. In UC, available data could not find a similar association (25, 40, 43).

Regarding disease location, differences between isolated ileal and colonic disease manifestation have been described. *Post-hoc* analysis of a placebo-controlled trial with certolizumab pegol showed higher probability of patients with colonic compared to isolated ileal disease to achieve clinical remission at week 6 of induction therapy (44). Several cohort studies also indicated better short-term and sustained clinical response to anti-TNF therapy in isolated colonic than in ileal CD (45, 46). Endoscopic and histologic healing were also more frequent in colon that the ileum after 1 year of adalimumab therapy in the EXTEND trial (47). For UC, there was no association between disease extend and probability of therapeutic induction and maintenance response to anti-TNF treatment (25, 27, 30).

### Disease Phenotype

Regarding the phenotypic manifestation, better short- and long-term response rates of anti-TNF therapy have been shown for non-stricturing and non-penetrating disease (Montreal Classification B1) in comparison to stenosing (B2) or fistulising disease (B3) (22, 48–51).

### Comorbidities

A recently published study showed that the presence of the comorbidities chronic obstructive pulmonary disease as well as extra-intestinal hepato-pancreato-biliary conditions were associated with primary non-response and myocardial infarction and skin disease were significantly associated with loss of response to anti-TNF treatment (52). Further studies will have to investigate these findings.

### Disease Severity

For disease severity, clearest data are available for UC. Here, anti-TNF therapy in severe disease showed diminished primary efficacy rates compared to treatment of less severe disease (25, 53–55). This might be due to the demonstrated fecal loss of anti-TNF through ulcerated intestinal mucosa into the stool of patients with high inflammatory burden (56). Another possible explanation might be that severe inflammation with high local TNF tissue concentrations could act as a sink for anti-TNF agents. This would explain why patients with high serum drug concentrations still fail to benefit from anti-TNF therapy, as insufficient tissue levels of anti-TNF are unable to neutralize heightened local TNF production (57).

### CRP, Fecal Calprotectin, Hemoglobin, Neutrophil-Lymphocyte Ratio, Albumin

A correlation between elevated C-reactive protein (CRP) levels and primary and sustained response to anti-TNF drugs has also been found in CD patients for all approved anti-TNF agents (41, 42, 49, 58–60). Analyses of the SONIC study have shown that elevated CRP levels were indicative of underlying inflammatory activity, thus predicting higher primary and long-term response rates than patients without inflammation (59). Nevertheless, not all CD patients with active disease exhibit elevated CRP-levels (61). In UC, higher anti-TNF induction and maintenance efficacy could be found in patients with low CRP-levels (21, 62).

Fecal calprotectin measurements have established themselves as surrogate measure for inflammatory activity in IBD (63). However, there have so far not been any conclusive results in relation to an association between fecal calprotectin levels and response to therapy (13).

Higher hemoglobin levels at baseline have only been shown to be associated with short- and long-term response to anti-TNF therapy in UC (53, 64, 65), but not CD (66).

One study reported that a high baseline neutrophil–to–lymphocyte ratio (cut-off value of 4.488) predicts secondary loss of response to infliximab treatment in UC patients (67).

Several studies have indicated that pre-treatment albumin levels correlate with primary response to anti-TNF therapy in UC, with lower levels showing worse response (29, 54, 64, 68). This might be due to diminished anti-TNF drug levels in hypoalbuminaemic patients (68).

### Previous Anti-TNF Exposure and Combination Therapy

There are several studies that have shown that previous anti-TNF therapy is associated with heightened probability of primary treatment failure and secondary loss of response of subsequent anti-TNF therapy (25, 43, 66, 69). A systematic review and meta-analysis reported that the efficacy of a second anti-TNF in CD patients was largely dependent on the cause for switching, as remission rates were higher in patients with previous anti-TNF intolerance (61%), compared with secondary (45%) or primary failure (30%) (70). Two randomized trial results underlined the primary benefit of concomitant immunomodulator therapy in infliximab treated IBD patients. In the SONIC trial, corticosteroid-free clinical remission at week 26 was seen in statistically significant more CD treated with azathioprine and infliximab, compared to those receiving infliximab or azathioprine alone (59). In the randomized SUCCESS trial in UC patients, corticosteroid-free remission at week 16 was achieved by more patients under infliximab and azathioprine treatment, compared with those receiving infliximab or azathioprine alone (71).

### Previous Surgery

Previous surgery in CD patients has been described as a negative factor for primary therapeutic response to anti-TNF therapy (18, 19), but this finding was not confirmed by other studies (22, 26, 48).

### Serological Antibody Markers

Antinuclear antibody (ANA) seropositivity has been associated with anti-TNF secondary non-response (72). Anti-OmpC positivity was associated with a lack of response to anti-TNF therapy at 1 year and increased likelihood of therapy discontinuation in UC patients (73). Low baseline levels of IgG antibodies against the pattern recognition receptors IFI16 were associated with clinical response to infliximab induction treatment in UC (74). Several studies tested the capacity of the serological marker perinuclear anti-neutrophil cytoplasmic antibodies (pANCA) to predict response to anti-TNF agents. A meta-analysis showed that pANCA negative patients had nearly a 2 fold higher response to anti-TNF therapy compared with patients who were pANCA positive. However, testing for pANCA positivity to predict non-response to infliximab therapy showed a sensitivity of only 25% and a specificity of 85%, leading to a positive predictive value of 41%, and a negative predictive value of 74%. These data indicate that pANCA testing are not applied in daily clinical practice for predicting response to therapy (75).

### Matrix Metalloproteinases

Loss of responsiveness might also be caused by heightened activity of matrix metalloproteinases in IBD non-responders, as they mediate proteolytic mucosal degradation of anti-TNF antibodies (76). Heightened clearance of TNF–anti-TNF antibody immune complexes through Fc receptor-mediated endocytosis and subsequent proteolytic degradation by the hyperactive reticuloendothelial system, might also contribute to non-response in UC patients (77).

## Pharmacogenomics

Genome-wide association studies (GWAS) have been able to identify susceptibility loci in IBD (78), and analyses of germline genetic variants have repeatedly been investigated for their predictive capacity in anti-TNF treated patients.

### Crohn's Disease

NOD2 which has been identified as a susceptibility gene for CD, did not show an association with primary response to infliximab treatment (79, 80). Missing association for primary response was also described for polymorphisms in the genes encoding TNFR1 and TNFR2 (81, 82). In patients with luminal CD, the −843 CC/CT genotype of the apoptosis inducing protein Fas ligand was associated with higher primary clinical response rates (75 vs. 38%; *p* = 0.002) to infliximab than patients with the TT genotype. Same was seen for patients with fistulizing CD (85 vs. 40%; *p* = 0.001). In addition, patients with the caspase-9 93 TT (*n* = 9) genotype all responded, in contrast with 67% (*n* = 147) with the CC and CT genotype (*p* = 0.04) (83). Subsequently, the author group then proposed an apoptotic pharmacogenetic index based on their pharmacogenetic study of apoptosis genes (Fas ligand −843 C/T, Fas −670 G/A and caspase-9 93 C/T) and clinical predictors as a model for prediction of low, medium, and high primary responses to the first infusion of infliximab in patients with CD (84). Further associations between genetic loci and primary response to anti-TNF therapy have been described for the IBD5 locus in CD (85). Another study indicated that single-nucleotide polymorphisms (SNPs) associated with genetically determined high activity of TLR5 among primary CD responders (86). Polymorphisms at the FCGR3A locus, encoding IgG Fc receptor IIIa, have been shown to be associated with a CRP decrease in primary response to infliximab in CD (87). This finding was confirmed by subsequent studies in CD (88, 89). The FCGR3A V158F polymorphism seems to be associated with anti-drug antibody formation in anti-TNF treated CD patients, correlating with dose intensification in these patients. Moreover, anti-drug antibody formation has been shown to be significantly associated with the HLA-DQA1^*^05 allele in CD patient, leading to heightened probability of secondary loss of response to anti-TNF monotherapy, necessitating the need for immunosuppressive combination therapy (90). CD patients with FCGR3A polymorphisms or HLA-DQA501 might therefore need combination therapy with immunomodulators and anti-TNF drugs in the subgroup to inhibit anti-drug antibody formation and subsequent loss of response. The autophagy related gene ATG16L1 was indicative for primary response to anti-TNF therapy in one study (91), but data from a subsequent study could not confirm this finding (92). Recently, response of 427 CD patients to their first anti-TNF therapy was characterized. Here, 15 risk alleles were associated with primary non-response, as these patients had a significantly higher genetic risk score. A combined clinical-genetic model more accurately predicted primary non-response, when compared with a clinical only model (0.93 vs. 0.70; *p* < 0.001) (23). Furthermore, the combination of two–risk genotypes, involving both apoptosis and the TNF region, was associated with primary anti-TNF non-response (93).

### Ulcerative Colitis

There was an association of homozygous high-risk (rs1004819, rs2201841, rs10889677m rs11209032, rs1495965) compared to low-risk (rs7517847m rs10489629, rs11465804, rs1343151) IL-23 receptor polymorphisms with primary response to infliximab therapy in UC patients (94). Another study identified eight alleles associated with primary non-response in UC. Here, a combined clinical-genetic model significantly more accurately predicted primary non-response compared with a clinical-only model. Importantly, genetic risk scores for primary non-response were not associated with infliximab levels or antibody formation (95). Unlike in CD, no association between primary response to anti-TNF therapy and the IBD5 locus could be found in UC (85). Another study indicated SNPs associated with genetically determined high activity of IL-12 and IL-18 levels among patients with UC were associated with primary non-response to anti-TNF treatment (86).

### Crohns's Disease and Ulcerative Colitis

Nuclear Factor kappa-light-chain-enhancer of activated B cells (NFκB) has been identified as a pivotal transcription factor in IBD pathogenesis (96) and polymorphisms in genes implicated in the NFκB-mediated primary response have been linked to anti-TNF treatment response in an IBD patient cohort study (97). Another study found that polymorphisms in genes involved in the regulation of the NFκB pathway (TLR2, TLR4, and NFKBIA), the TNF-α signaling pathway (TNFRSF1A), and other cytokine pathways (NLRP3, IL1RN, IL18, and JAK2) were associated with primary response to anti-TNF therapy in IBD patients (98).

In a recently published study, two successfully replicated genetic loci (rs116724455 in TNFSF4/18, rs2228416 in PLIN2) and four with suggestive evidence were found, that increased predictability of an exploratory risk model for primary non-response from initially 0.72 (clinical predictors) to 0.89 after adding the genetic predictors (99). A systematic review and meta-analysis of available studies with at least 100 BD patients included, indicated that apart from afore mentioned FCGR3A, polymorphisms in TLR4, TNFRSF1A, IFNG, IL6, and IL1B genes were also significantly associated with heightened primary response, whereas TLR2 and TLR9 variants with reduced response (100). Altogether, the mentioned studies indicate the potential of gene polymorphisms to predict response to anti-TNF therapy, but further large trials are needed to validate the mentioned findings.

## Intestinal Microbiome

Several studies have indicated that the gut microbiome and its interaction with the mucosal immune system is critically involved in driving the inflammatory reaction in IBD patients (101). Dysregulation of the microbiome has been reported in IBD patients with reduced diversity and temporal instability of the dominant taxa compared with healthy controls (102).

### Microbiota Changes

First studies investigated a possible relationship between specific changes in the microbiota and prediction of clinical response to anti-TNF therapy. In a prospective study in pediatric IBD patients, higher amounts form the groups of *Bifidobacterium ssp., Eubacterium rectale, Clostridium colinum*, uncultured *Clostridiales*, and *Vibrio* and lower presence of *Streptococcus mitis* were found in primary responders than in non-responders (103). In another study, besides the antimicrobial peptides defensin 5 and eosinophilic cationic protein, lower dysbiosis indices and higher abundance of *Faecalibacterium prausnitzii* at baseline were also found in primary responders compared to non-responders to anti-TNF treatment (104).

### Metabolomic Predictors

As differences in the composition of the intestinal microbiota have been linked to changes in metabolite concentrations, recent studies also focussed on possible metabonomic predictors of primary response. Total metabolic exchange was significantly disrupted at baseline in fecal samples from IBD non-remitters. Butyrate and substrates involved in butyrate synthesis, such as ethanol or acetaldehyde, were less frequently exchanged among bacterial communities from patients who did not show primary therapeutic efficacy in response to anti-TNF therapy (105). Disturbances in an association network containing taxa of the *Lachnospiraceae* and *Ruminococcaceae* families, typically producing short chain fatty acids, were shown to characterize poor primary responses to treatment with anti-TNF-α therapeutic antibodies (106). A recently published prospective, longitudinal cohort study in CD patients identified metabolic profiles, which were predictive of primary anti-TNF non-response with alterations in bile acid, amino acid, and lipid pathways (107).

## Immunological Markers

### Proteomics

Large-scale detection, identification and characterization of proteins is a another domain of biomarker research in IBD (108). So far, only few studies have evaluated the capacity of proteomics for the prediction response to treatments. Serum proteomic profiling by surface enhanced laser desorption ionization time of flight-mass spectrometry (SELDI-TOF-MS) was applied in CD patients prior initiation of infliximab treatment. The author group found an association between platelet metabolism, in particular platelet aggregation factor four, and primary response to infliximab (109).

In another study, serum samples were subjected to two-dimensional gel electrophoresis, and after evaluation of densitometrical data, protein spots exhibiting differential expression among the groups, were further characterized by matrix-assisted laser desorption ionization time-of-flight mass spectrometry (MALDI-TOF-MS). The proteins apolipoprotein A-I, apolipoprotein E, complement C4-B, plasminogen, serotransferrin, beta-2-glycoprotein 1, and clusterin were found to be up-regulated in the primary non-responder and responder groups, whereas their levels displayed no changes in the remitters group when compared to baseline samples. Additionally, leucine-rich alpha-2-glycoprotein (A2GL), vitamin D-binding protein (VTDB), alpha-1B-glycoprotein (A1BG), and complement C1r subcomponent (C1R) were significantly increased in the serum of primary remitters.

The label-free physiological intermolecular modulation spectroscopy (PIMS) was applied in peripheral blood mononuclear cells of IBD patients to identify responders to infliximab treatment. PIMS takes into account a combination readout based on changes in the resonance of water molecules and macromolecular conformation. PIMS data predicted primary response to anti-TNF therapy with an accuracy of 96% (110). All mentioned pioneering proteomic pilot study data require validation in larger cohort of patients.

## Blood Markers

### Cytokines

There are also several studies that primarily assessed the predictive value of blood parameters regarding prediction of response to anti-TNF therapy. High serum IL-1β concentrations were associated with lower primary clinical remission to infliximab in CD (111). IL-8 concentrations at baseline were higher in primary non-responders compared to responders in CD patients treated with infliximab. Multiple logistic regression identified TNF/CRP ratio at baseline as predictive for primary non-response to infliximab at week 14 (112).

Another study investigated the *in vitro* capacity of anti-TNF antibodies on cultured peripheral blood cells to suppress T cell surface receptor expression and cytokine release. The study found that anti-TNF suppressed the expression of CD25 on T cells and secretion of interleukin 5, to a higher degree in UC primary responders than in non-responders. A created prediction model was subsequently tested in a validation cohort. Correct classification of future therapy response was here achieved in 91% of the cases (113). In UC patients, primary anti-TNF non-responders had significantly increased TNF, IFNγ, IL-1β, and IL-10 levels compared to responders. Non-responders also demonstrated significantly lower TNF and IL-1β production by cultured peripheral blood mononuclear cells to various Toll-like receptor stimulation compared to responders, as well as reduced TLR9-induced IL-6 and TLR-3,−4,−8, and−9-induced IL-10 (114).

A recently published study investigated TNF production by cultured and lipopolysaccharide stimulated peripheral blood mononuclear cells from IBD patients prior to infliximab therapy initiation. Primary responders demonstrated significantly higher TNF and IL-6 production than non-responders. In CD patients, a certain threshold of TNF levels identified responders with 100% sensitivity and 82% specificity. This finding was confirmed in multivariate analysis. The percentage of TNF-positive cells was higher in CD14+ monocytes compared to lymphocytes after stimulation (115).

### Vitamin D

Recent studies investigated a possible correlation between vitamin D levels and clinical response to infliximab therapy. Here, low baseline vitamin D concentration was associated with heightened probability of primary clinical remission at week 14 in CD patients (116). Another study in IBD patients, found a significant link between deficiency of vitamin D and the presence of ANA, which were found to be associated with failure to anti-TNF therapy and also reported as significant risk factors for anti-TNF induced adverse events associated with anti-TNF therapy (72).

## Tissue Markers

The analyses of gene expression via RNA sequencing in inflamed tissue or intestinal immune cells of patients have enlarged our insights into the immunopathogenesis of IBD.

### Different Gene Signature Profiles

A study in patients with colonic CD, identified a gene signature profile composed of TNFAIP6, S100A8, IL11, G0S2, and S100A9, which predicted primary infliximab response with 100% accuracy (117). A subsequent study performed by another group in their cohort of CD patients supported the role of the reported expression signature as predictive for primary anti-TNF outcome (118). High baseline IL13RA2 levels were associated with lack of mucosal healing in anti-TNF treated CD patients. The authors also showed TNF-driven pathways were significantly enriched in primary non-responders to infliximab and linked to increased mucosal IL13RA2 expression (119). GATA3 expressing lamina propria CD4+ T lymphocytes were increased in anti-TNF endoscopic primary non-responders compared to responders in CD patients (120).

One of the first studies to investigate the predictive capacity of gene expression profiles in UC patient samples and primary response to subsequent anti-TNF therapy was undertaken in 2009. Here, colonic tissue transcriptomics in biopsy samples that were taken prior to initiation of infliximab therapy in two cohorts of UC patients led to the identification of a five-gene signature consisting of osteoprotegerin, stanniocalcin-1, prostaglandin-endoperoxide synthase 2, IL-13 receptor alpha 2 (IL13RA2), and IL-11, that are all involved in the adaptive immune response. This panel of genes separated responders from non-responders with 95% sensitivity and 85% specificity (121).

Other studies investigated cytokine transcript changes in pre-treatment mucosal biopsies. One study in UC patients reported higher expression of genes encoding IFN-γ and IL-17 in the mucosa of anti-TNF therapy primary responders compared to non-responders (122). On the other hand, another study showed that UC week 14 responders had lower mucosal mRNA expression of interleukin IL-1β, IL-17A, IL-6, and IFN-γ than primary non-responders. In a study with CD patients, high expression of IL-17 and IL23 was found in infliximab responders in comparison to primary non-responders (123).

In a study with UC patients, mucosal healing upon initiated anti-TNF therapy was associated with lower pre-treatment mucosal expression of transcription factor Th1-Tbet and higher expression of Th17-Rorc (124) in primary responders. Furthermore, GATA3 expressing lamina propria CD4+ T lymphocytes were increased in anti-TNF endoscopic primary non-responders compared to responders in CD patients (120). In a recently published study, the authors used a colonic 13-gene transcript panel that had previously shown an association with efficacy of anti-TNF therapy, to predict therapeutic response to golimumab in UC patients. The baseline gene expression signature predicted mucosal healing with a sensitivity of 87%, but with a specificity of only 34%, indicative of a high false positive rate. The gene expression signature was not able to identify patients who would achieve primary clinical response or clinical remission (125).

### TREM-1

Another study found increased baseline presence of mucosal plasma cells and inflammatory macrophages in colonic biopsy samples from IBD patients who did not primarily respond to anti-TNF therapy. Abundance of inflammatory macrophages were associated with increased expression of the triggering receptor expressed on myeloid cells (TREM-1), chemokine receptor type 2 (CCR2), and chemokine ligand 7 (CCL7). Blood gene expression analysis of an independent cohort, identified TREM-1 downregulation in primary non-responders at baseline, which was predictive of clinical response with an AUC of 94%. This was also one of the few studies, where results were validated in independent cohorts (126). Strikingly, another study described downregulated TREM1 expression in the blood of IBD patients with endoscopic remission upon anti-TNF therapy (127). These contrary findings regarding TREM-1 expression in primary responder and non-responders to anti-TNF therapy, although regarding differing endpoints consisting of, respectively, clinical and endoscopic parameters, demonstrate the need for further studies.

### TNF

Several studies have shown that TNF levels are markedly increased in the serum and intestinal tissue of IBD patients (128), centrally regulating the intestinal inflammatory process in multiple ways. Here, studies have shown that the transmembrane precursor protein mTNF expressed on immune cells rather than soluble TNF (sTNF) is the pivotal factor in perpetuating the inflammatory reaction in IBD, thereby also representing the decisive target for effective anti-TNF therapy (129, 130). Induction of mucosal T cell apoptosis has been described as the main mechanism of action of efficacious anti-TNF treatment in IBD, as intestinal T cell resistance to apoptosis is important for sustaining chronic intestinal inflammation (131, 132). Application of anti-TNF drugs to disrupt the costimulatory interaction between mTNF on CD14+ macrophages and tumor necrosis factor receptor 2 (TNFR2) on T cells from the mucosa of patients with IBD has been shown to induce T cell apoptosis (133). Thus, a correlation between the level of mucosal TNF expression and the efficiency of the TNF antibody directed against it was subsequently analyzed.

One study harnessed the diagnostic method of molecular endoscopy (134–136), to prospectively analyse a correlation between mucosal mTNF expression and effectiveness of anti-TNF therapy in CD patients. Mucosal mTNF expressing cells were visualized *in vivo* by topical application of a fluorescent anti-TNF antibody in conjunction with confocal laser endomicroscopy (CLE) during a conventional colonoscopy procedure. Patients with high numbers of intestinal mTNF+ cells showed statistically significantly higher primary clinical response rates at week 12 than patients with low numbers mTNF+ cells. Patients with high mTNF expression rates also reached endoscopic remission more often over a follow-up period of 1 year (137).

One study in UC patients found an inverse and independent association between pre-treatment mucosal TNF expression levels and primary clinical and endoscopic remission of infliximab treatment (138).

## Mechanisms of Resistance to Anti-TNF Therapy

Recently, the concept that changes in the composition of immune cell infiltrates in response to therapeutic pressure lead to molecular resistance to the applied drug has been introduced to the IBD filed (10). An improved understanding of molecular resistance is essential to optimize personalized treatment in IBD. First studies have indicated mechanisms that drive primary resistance to biological therapy in IBD.

### IL-23 and IL23R+TNFR2+ T Cells

A recent study indicated that excessive IL-23 production by CD14+ gut macrophages is one of the main drivers of evasion of apoptosis upon anti-TNF antibody therapy in CD non-responders. This results in the expansion of apoptosis-resistant IL23R+TNFR2+ T cells that mediate resistance to anti-TNF therapy (139).

### OSM

One of the best validated studies indicating activation of a TNF-independent signaling pathway in anti-TNF resistant patients (10), was based on analyzing mRNA expression levels in mucosal biopsies taken prior anti-TNF therapy. The study associated oncostatin M (OSM) with primary failure to anti-TNF therapy in IBD patients. These data were found by analysis of over 200 patients with IBD, including two well-described cohorts from phase three clinical trials of infliximab and golimumab. Fittingly, in an animal model of anti-TNF-resistant intestinal inflammation, genetic deletion, or pharmacological blockade of OSM significantly diminished colitis activity (140). Further studies also associated elevated plasma OSM and nCD64 expression in pediatric CD patients with poor biochemical outcomes (<50% reduction in FC from baseline at week 12) to infliximab treatment (141). Another recent study demonstrated that serum OSM levels were significantly lower in CD patients with mucosal healing at week 54 upon infliximab treatment than in patients not achieving this endpoint (142).

### IL7R Depending Signaling Pathway

Another study elucidated heightened expression of the IL7R and the IL-7 dependent signaling pathway in the inflamed colon of IBD patients non-responsive to anti-TNF therapy. The IL-7R signaling specifically regulates effector but not regulatory T cell homing to the gut by controlling alpha4 and beta7 integrin expression, thereby implicating blockade of the IL-7R as a novel therapeutic option in IBD (143).

### IL-22BP

A recent study delineated the pathogenic role of the IL-22 binding protein (IL-22BP) in IBD. Data of the study suggested that efficacious anti-TNF treatment may block IL-22BP expression by intestinal T cells, enabling IL-22 induced mucosal healing. Correspondingly, T cell derived IL-22BP was not downregulated in anti-TNF primary non-responders, thereby suggesting that direct targeting of IL-22BP might represent an effective treatment option (144).

### GIMATS Module

Recently, single-cell analysis of inflamed intestinal tissue from CD patients depicted that cellular heterogeneity contributes to anti-TNF treatment resistance. A unique cellular composition that consisted of IgG plasma cells, inflammatory mononuclear phagocytes, activated T cells, and stromal cells, which was classified as the GIMATS module, in active lesions was associated with failure to achieve durable remission upon anti-TNF therapy. Results of the study suggest that combining anti-TNF antibodies with drug targets that block key nodes in the GIMATS response may represent an opportunity to overcome anti-TNF resistance in patient with high GIMATS expression. Here, inflammatory macrophage-derived stimulatory mediators such as IL-1ß or OSM were implicated to trigger stromal activation in GIMATS^high^ lesions (145).

## Conclusion

Although significant amount of scientific data has been collected to identify a reliable biomarker for prediction of therapeutic response to anti-TNF treated IBD patients, none of them have entered daily clinical practice as a decisive tool to enable an individualized therapeutic approach. Even 20 years after introduction of this substance class to our therapeutic armamentarium, there is still the unmet need for a reliable marker that would allow a more rational application of anti-TNF treatment in IBD. The currently applied clinical practice of randomly commencing a biological treatment and assessing response to therapy several weeks after initiation is coupled with progression of tissue damage in non-responders, risk of systemic side-effects, and substantial health-care costs of an inefficient therapy. Prediction of therapeutic response would allow optimization of the risk/benefit ratio of anti-TNF inhibition in IBD.

The potential of molecular stratification of patients to enable a personalized treatment approach (146) is best visible in pediatric patients with early onset IBD, which is driven by high penetrance alleles or by the dysfunction of a single gene (147, 148). Here, identification of monogenic IBD forms led to initiation of specific targeted therapies that were able to ameliorate intestinal inflammation (149). However, personalized treatment of polygenic IBD has so far not been able to be based on genetic information alone.

Current data demonstrate that response to anti-TNF therapy may be influenced by many factors that consist of disease-related and clinical characteristics, biochemical markers, blood and stool derived parameters, pharmacogenomics, microbial, and metabolic factors, as well as local mucosal factors. These studies are important contributions toward identification of a clinically applicable biomarker.

A suitable biomarker should ideally be non-invasively assessed, validated, rapidly quantifiable, inexpensive to measure, easily reproducible, and importantly not influenced by various confounders. Future trials that aim to validate a predictive biomarker of response must therefore also take into account other factors that have been shown to influence the efficacy of biological therapies, reflecting the complexity of such an approach. Nevertheless, interpretation of these findings must also take into account possible decisive influence of pharmacological factors, as a recently published prospective cohort study in CD patients (PANTS study), demonstrated that the only factor independently associated with primary anti-TNF non-response was low drug concentration at week 14 (24). Future studies should therefore also implement measurement of anti-TNF trough levels in the trial design to ideally identify predictive factors independent of serum drug levels. There is sufficient evidence that implies that pharmacokinetic factors alone are rather insufficient to reflect non-response, as even patients with sufficient drug levels fails to benefit from anti-TNF therapy, strongly implying mechanistic reasons for failure (10, 150). Trials should be performed separately in each IBD entity with clear definition of the studied end-point that defines response to therapy, which ideally should include endoscopic outcomes (151). Potential biomarkers need prospective validation in multi-center studies with large cohorts of patients and should incorporate short-term and long-term observations. Endoscopic, clinical, and laboratory baseline characteristics should ideally be evenly distributed when comparing responders and non-responders to therapy, to exclude influence of confounding factors. As reasons for non-response are possibly multifactorial, studies should also not restrict themselves to only analyzing one factor, but rather incorporate many markers and investigate in how far they might even influence each other, especially for molecular markers. This is best visible in the area of transcriptomic studies, which have helped us to understand disease-associated changes, but one must be aware that the functional relevance of these findings are unclear, as they do not take into account potential post-translational modifications. These studies should therefore ideally be backed up by corresponding protein quantification.

**Figure 1:**
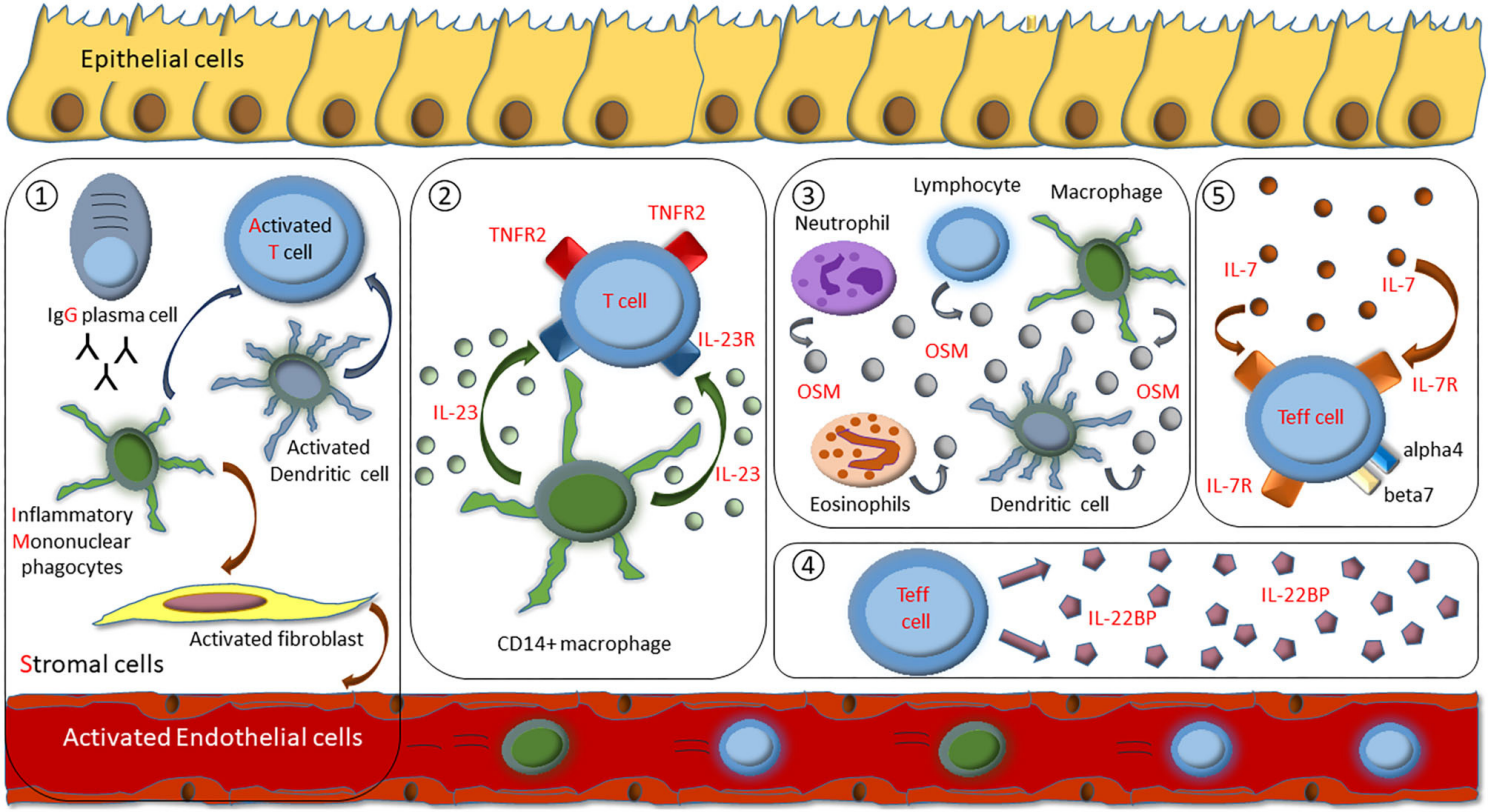
Overview of identified mechanisms of molecular resistance to anti-TNF therapy in IBD patients. (1) Cellular composition of IgG plasma cells, inflammatory mononuclear phagocytes, activated T cells, and stromal cells (GIMATS module). (2) Excessive IL-23 production by CD14+ gut macrophages drive expansion of apoptosis-resistant IL23R+TNFR2+ T cells. (3) Overexpression of intestinal oncostatin M (OSM). (4) Overexpression of T cell derived IL-22BP. (5) Heightened expression of the IL-7R dependent signaling pathway that specifically regulates effector T cell homing to the gut by controlling alpha4 and beta7 integrin expression.

It is reasonable to expect that exposure to anti-TNF inhibitors induces emergence of TNF-independent inflammatory pathways that mediate resistance to anti-TNF therapy. Recent insights into mechanisms that drive resistance to anti-TNF therapy provide a comprehensive cellular and molecular basis to overcome this process with novel therapeutic approaches, like inhibitory agents targeting IL-23, OSM, IL-7R, IL-22BP, or IL-1ß (Figure 1). These insights might help us to not only understand mechanistic reasons for anti-TNF failure, but could also lead the way to tailor subsequent treatment options for the benefit of the patient.

In summary, currently no single marker fulfills all criteria for being an appropriate prognostic indicator for response to any anti-TNF treatment in IBD, and therefore the suggested biomarkers appear of limited clinical utility. Upcoming research should aim to develop a predictive model that incorporates all relevant factors derived from ongoing research, as indicated in our narrative review, to establish a reliable and validated tool that allows us to open new avenues for personalized medicine. The development of predictors of anti-TNF response is of central clinical importance and might be essential to their future use in the therapeutic algorithm of treating IBD patients.

## Author Contributions

All authors have made substantial contributions to all of the following: (1) the conception and design of the study, or acquisition of data, or analysis and interpretation of data, drafting the article, revising it for important intellectual content, and final approval of the version to be submitted.

## Conflict of Interest

RA has served as a speaker, or consultant, or received research grants from AbbVie, Biogen, Boehringer Ingelheim, Celgene, Dr. Falk Pharma, Ferring, InDex Pharmaceuticals, Janssen-Cilag, MSD Sharp &, Dome, Pfizer, Roche Pharma, Samsung Bioepis, Takeda. MN reports research grants and/or personal fees from Abbvie, MSD, Takeda, Boehringer, Roche, Pfizer, Janssen, Pentax and PPD. BS, has served as Consultant for Abbvie, Boehringer, Celgene, Falk, Janssen, Lilly, Pfizer, Prometheus, Takeda, and received speaker's fees from Abbvie, CED Service GmbH, Falk, Ferring, Janssen, Novartis, Takeda (BS served as representative of the Charité).
